# Engagement of the motor system in position monitoring: reduced distractor suppression and effects of internal representation quality on motor kinematics

**DOI:** 10.1007/s00221-018-5234-2

**Published:** 2018-03-15

**Authors:** Christina J. Howard, Hayley Boulton, Emily Brown, Craig P. A. Arnold, Matthew K. Belmonte, Suvobrata Mitra

**Affiliations:** 1 Room 103, Taylor Building, Nottingham Trent University, 50 Shakespeare Street, Nottingham, NG1 4FQ UK; 2 Royal Holloway, University of London, London, UK; 3 The Com DEALL Trust, Bangalore, India; 4 Centre for Autism, School of Psychology and Clinical Language Sciences, University of Reading, Reading, UK

**Keywords:** Divided attention, Motor control, Multiple object tracking, Spatial vision, Position monitoring

## Abstract

The position monitoring task is a measure of divided spatial attention in which participants track the changing positions of one or more objects, attempting to represent positions with as much precision as possible. Typically precision of representations declines with each target object added to participants’ attention load. Since the motor system requires precise representations of changing target positions, we investigated whether position monitoring would be facilitated by increasing engagement of the motor system. Using motion capture, we recorded the positions of participants’ index finger during pointing responses. Participants attempted to monitor the changing positions of between one and four target discs as they moved randomly around a large projected display. After a period of disc motion, all discs disappeared and participants were prompted to report the final position of one of the targets, either by mouse click or by pointing to the final perceived position on the screen. For mouse click responses, precision declined with attentional load. For pointing responses, precision declined only up to three targets and remained at the same level for four targets, suggesting obligatory attention to all four objects for loads above two targets. Kinematic profiles for pointing responses for highest and lowest loads showed greater motor adjustments during the point, demonstrating that, like external environmental task demands, the quality of internal representations affects motor kinematics. Specifically, these adjustments reflect the difficulty of both pointing to very precisely represented locations as well as keeping representations distinct from one another.

## Introduction

We used the position monitoring task to assess the extent to which increasing engagement of the motor system can affect the way we can attend to spatial locations. This is a variant of the multiple object tracking (MOT) task (Pylyshyn and Storm 1988) where participants attempt to keep track of moving target objects amongst distractors. Pylyshyn and Storm originally proposed a set number of mental ‘pointers’ with which we keep track of these positions. A more probable model in light of recent evidence (Alvarez and Franconeri 2007; Howard and Holcombe 2008) is a continuous resource that can be focussed on fewer targets or spread more thinly over a greater number. We here ask whether this spatial tracking resource could wholly or partially reside in the mechanisms responsible for motor preparation. Several lines of research point towards this possibility: an unrelated finger-tapping task interferes with MOT (Trick et al. 2006) and individuals with Parkinson’s disease, traditionally thought of as a motor disorder, are impaired on tracking tasks (Norton et al. 2016). An interactive version of MOT requiring finger movements to control objects appears to yield higher capacity estimates than traditional MOT (Thornton et al. 2014) and tracking appears to be sensitive to the postural demands of standing versus sitting (Faubert and Sidebottom 2012). Further, some have argued that athletes show superior tracking (Faubert 2013; but see; Memmert et al. 2009). Much more generally, Gibson’s (1979) conceptualisation of affordances places vision as a means to perceiving the possibilities for action in the environment—a view that would predict tight coupling between tracking tasks and motor processing.

There is also a large degree of overlap between brain areas responsible for tracking and motor imagery. Parietal areas are recruited during tracking, particularly intraparietal sulcus (IPS) (Blumberg et al. 2015; Culham et al. 1998; Howe et al. 2009; Jahn et al. 2012; Jovicich et al. 2001). IPS is also involved in imagined movements (de Lange et al. 2006; Grèzes and Decety 2001) as are posterior parietal (de Lange et al. 2005) and inferior parietal areas (Guillot et al. 2009). It also appears that the tracking resource, like motor control, is somewhat independent in the two hemispheres (Alvarez and Cavanagh 2005; Chen et al. 2013; Holcombe et al. 2014). Together, these findings point to the possibility that motor imagery or rehearsal is used to support spatial attention, particularly if a motor response is required.

The motor control system is known to be predictive (Davidson and Wolpert 2005), a capacity evidenced in our ability to intercept moving targets (Smeets and Brenner 1995; Soechting et al. 2009). In the case of manual interception, sensory feedback regarding the current location of one’s own hand as well as the position of the target is typically available online during the motor response, although continuously looking at the target is not always necessary in order to successfully intercept that target (Land and McLeod 2000). Whether or not visual information is available during the motor output itself, the motor control system is therefore a viable candidate for processes that support the ability of the visual system to keep up to date with dynamic stimuli in order to prepare for possible action. Therefore, it seems sensible to ask whether explicitly increasing the engagement of the motor system may facilitate position tracking in terms of spatial precision.

A related reason that motor contributions may facilitate position tracking is to assist in processing of temporal aspects of stimuli such as extrapolatory processes. In position monitoring and tracking tasks, some report extrapolation of the representation of moving targets (e.g. Atsma et al. 2012; Iordanescu et al. 2009), whereas others report attention lagging behind the stimulus (Lukavský and Děchtěrenko 2016). The position monitoring variant of the MOT task (Howard and Holcombe 2008; Howard et al. 2011, 2017) is uniquely able to test for the presence of perceptual lag, that is to say the tendency of participants to report positions from the recent past instead of the most up-to-date position of targets. This sensitivity to perceptual lag arises because this method, beyond probing the precision of position representations, can also assess the similarity between reported positions and the actual positions of targets at different time lags. An alternative to the perceptual lag analysis method is the angular error analysis (Iordanescu et al. 2009; Howard et al. 2011) in which each response is assessed in terms of where it falls relative to the final heading of the queried target. These perceptual lag and angular error measures are an indicator of the extent to which people are able to keep up-to-date representations about the changing visual world. Although attention must operate continuously while the stimulus is displayed, participants must make their response after the stimuli have disappeared. Therefore participants must form a representation of the target’s final position to be briefly stored offline while the response is being prepared. This method differs from traditional MOT tasks in that objects are often presented in separate areas of the display rather than crossing each other’s paths. This spatial separation enables individual targets to be queried by means of post-cueing one of the areas of the screen. Since the motor system has been demonstrated to be predictive under other circumstances as discussed above, we investigated whether engaging the motor system more directly might affect the ability of the visual system to keep up-to-date with changes in the environment. We asked participants to indicate the final position of one of the targets, responding either by mouse click or, to heighten motor engagement, by pointing to the last perceived position on the screen. It should be noted that in both cases, the response occurs after the stimuli have disappeared and hence the task is relatively offline compared to interception tasks, for instance. For these reasons, we predicted that increasing engagement of the motor system (by means of contrasting the pointing response condition with the mouse click condition) would not only facilitate position monitoring and potentially reduce load effects, but also decrease the magnitude of perceptual lag and increase the proportion of forwards-biased angular errors (or equivalently, increase extrapolation and reduce the proportion of backwards-biased angular errors).

In the motor control literature, it has been shown that the precision required for motor responses such as grasping and pointing directly affects the kinematics of the motor response. Specifically, demand for greater precision, for example when interacting with a physically small target, results in longer response times and extended duration of motion after peak velocity has been reached (Fitts 1954). To assess the generality of this claim, we additionally investigate how the precision of the representation of a no-longer-visible pointing target affects the pointing trajectory, since this relationship has not, to our knowledge, been investigated before. Others have previously shown that internal representations can guide motor responses, for example during memory-guided reaching (Heath 2005; Heath et al. 2004), memory-guided pointing (Wu et al. 2010) and reciprocal tapping tasks (Binsted et al. 2006). However, none to our knowledge have investigated the role of precision and noise in these internal representations in the guidance of motor responses. It has been shown that the number of attended targets during tracking of this kind is inversely related to the precision of their representations (Howard and Holcombe 2008; Howard et al. 2011, 2017). Therefore, lower loads should be associated with internal representations of greater precision, and load thus can serve as a proxy to assess the effect of the precision of the pointing target’s representation on the pointing motor trajectory during a position monitoring task. Because the response occurs after all stimuli have disappeared and one target is queried, only one representation remains task-relevant at the time of the response and for lower loads, this will have been represented more precisely, rendering the pointing target as a smaller area of space than it would be at higher loads, when representations are more spatially diffuse. We therefore predicted that, if the precision of internal representations acts in the same way as the precision of visible targets, then we should see more difficulty in pointing responses at lower loads compared to higher loads. Greater difficulty of the pointing response should be reflected in greater early adjustments to the pointing trajectory and longer periods of deceleration in the final moments of the trajectory.

By varying the extent of involvement of the motor system under different attention loads, we are able to answer three important questions: first, whether or not increasing engagement of the motor system facilitates position monitoring and reduces load costs; second, whether this increased engagement of the motor system enables the visual system to reduce perceptual lag and trigger more extrapolatory processes; third, whether increasing the precision of internal representations (at lower loads) increases difficulty of the motor output, just as has been previously shown for high precision physical targets.

## Method

We used a position monitoring task in which participants attended to between one and four moving target discs and then indicated the final perceived position of one queried target. In two different conditions, these position reports were made by either using a mouse click or by pointing to make a response. In the mouse click condition, participants used a mouse with their right hand to move a cursor and then click on the final perceived position of the queried target. In the pointing version, they used their right index finger to point at the final perceived position of the queried target.

### Participants

Twenty-seven participants (10 males and 17 females) aged between 18 and 32 years (mean 23 years) took part in this experiment. The protocol was approved by the Nottingham Trent University College of Business, Law and Social Sciences Research Ethics Committee. Participants were recruited from those responding to the advertisement specifications of being right handed, with normal or corrected-to-normal vision and had no self-reported neurological conditions. In addition, they were required to answer ‘right’ to a minimum of 6/10 items on the Edinburgh handedness inventory (Oldfield 1971) and ‘left’ to no more than one of these ten items.

### Apparatus

A two-sensor Codamotion CX1 motion-tracking system using Odin software (Charnwood Dynamics, Rothley, Leicestershire, UK) was used to capture participants’ movements. A set of Codamotion active markers recording at 100 Hz was attached to the participant at the hip and on the right hand on the side of the right index finger to record their positions in 3D space in the laboratory. Movement data from the hip were recorded to assess any effects of cognitive load on postural maintenance and to account for any contamination of apparent pointing trajectories by body motion. The PsychoPy script controlling stimulus presentation sent triggers at the start and end of each block to synchronise recording with the Odin system.

Before each experimental condition, participants completed a calibration stage to register the position tracking readings from the sensor on the finger and the position of the mouse cursor to the appropriate positions in screen co-ordinates. During the practice stage at the start of the session, participants also took part in practice versions of each of the two types of calibration sessions (pointing and mouse click) which were identical to the real calibration sessions. Both calibration sessions displayed a series of black 1.2° diameter target discs on a grey background (27.5 cd/m^2^) whose positions participants either pointed to or indicated using a mouse click. Four presentations of five disc positions appeared in a randomised order. The five potential positions were central presentation and 33.30° diagonally to the upper right, upper left, lower right or lower left of fixation. In the mouse click version, each time a disc appeared the participant was instructed to move a 1.7° white cursor disc using the mouse to click on the target disc position as accurately as possible. This mouse click triggered the disappearance of the disc, after which the next calibration disc immediately appeared. Participants were asked to make their responses quickly and without too much deliberation. The pointing version of the calibration phase was identical, except that responses were made by pointing directly with the index finger of the right hand at the calibration disc and then clicking the mouse in the left hand at the moment that the participant felt that they were pointing at the calibration disc. When the participant returned their right finger to the keypad, the next calibration point appeared.

### Procedure

Each participant took part in eight blocks of 16 trials of each of the two response conditions—mouse click responses or pointing responses, resulting in a total of 128 trials in each response condition. The order in which participants completed each of the two conditions was randomised. Within each block, participants completed 4 trials in each of four attention load conditions attending to either one, two, three or all four of four discs displayed. The order of these attention load conditions was fully randomised within blocks.

Before participating, participants were given 12–16 practice trials of each of the two response type conditions until they felt comfortable performing the task. The order in which each participant participated in these two types of practice (pointing, mouse click) was the same as the order in which that participant took part in the two experimental conditions. Participants stood barefoot with their feet hip distance apart and directly facing the screen at a distance such that, with their right arm held horizontally in front of them, their clenched fist just touched the screen. This distance was chosen since it enabled participants to subsequently extend their right index finger to reach the screen without exceeding maximum extension of the elbow nor necessitating leaning the body forwards. A stand was placed in front of them at hip height and both this stand and the screen were midline-aligned to the standing posture of the participant. Stimuli were presented in PsychoPy (Peirce 2007) and back-projected at 60 Hz using a Philips Picopix PPX4835 projector onto a 100 × 100 cm screen in a dimly lit room with a projection area measuring 88 cm wide × 61 cm high. Participants stood at a distance that, on average, placed the screen 62 cm in front of their eyes.

**Fig. 1:**
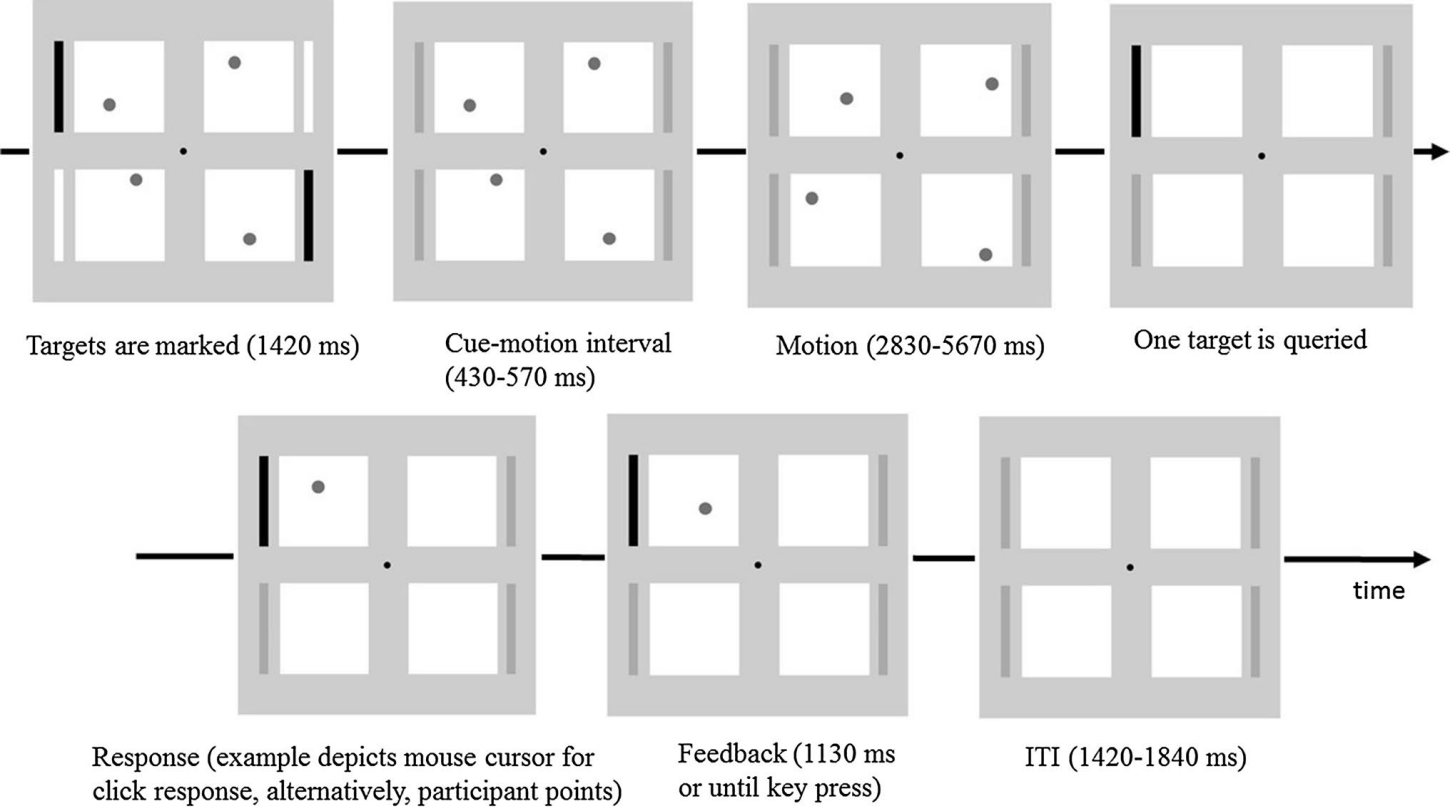
Representative trial timeline. On this two-target trial, the target cues are black and the participant makes their response using a mouse click. After the motion period (3rd panel from left, top row), all four discs disappear and the participant is prompted to report the final perceived position of the queried target (4th panel from left, top row). They are subsequently presented with feedback in the form of the veridical final position of the queried target in the position it had occupied at the moment before it disappeared. On this trial, the reported final position was above and to the left of the veridical final position of the queried target and thus the error magnitude was approximately 8°

Participants were instructed to fixate a central black fixation point throughout (0.7° diameter, 9.6 cd/m^2^). On every trial, four white areas (24.3° width and 19.0° height, 57.5 cd/m^2^) were presented to the upper left, upper right, lower left and lower right of fixation such that their centres were 14.8° from fixation (the vertical distance between their centres and the horizontal midline was 11.5° and the horizontal distance between centres and vertical midline was 11.3°), against a light grey background (27.5 cd/m^2^) (see Fig. 1). Four placeholder bars (1° × 24.3° and with their inner edges 2.1° from outermost edge of movement areas, 21.25 cd/m^2^) were constantly visible. On every trial, one stationary dark grey disc (1° diameter, 16.5 cd/m^2^) appeared in each area and the discs’ starting positions within each quadrant were randomly determined. On the same frame as the appearance of the discs, either one, two, three or four of the placeholders turned white (57.5 cd/m^2^), or black, (9.6 cd/m^2^ in 50% of randomly determined participants) for 1420 ms to signal the identity of the target disc or discs on that trial. The cues then offset and motion of the discs commenced after a variable delay uniformly distributed between 430 and 570 ms.

All four discs then underwent a period of random motion and participants attempted to keep track of the changing location of the target discs. Each disc’s initial velocity was determined by randomly selecting a horizontal and vertical component of motion between − 14.68 and 14.68°/s (negative values correspond to leftward or downward motion components) with the constraint that no total resultant speeds could be slower than 11.93°/s. Discs rebounded off boundary walls but otherwise continued with constant speed and direction. The duration of this motion phase was randomly selected on each trial between 2830 and 5670 ms.

After the motion phase, all four discs disappeared, and one target was immediately queried. This disc was queried by means of the same brightening (or darkening) as was used for the initial cueing of targets, but in this case for one target only. On all trials, participants then indicated the perceived final position of the queried disc, either by means of a mouse click or by pointing. Participants were asked to make their response ‘quickly and without too much deliberation’ and were not explicitly instructed to maintain fixation during this response phase.

During mouse click trials the mouse was placed centrally on the stand, with a keypad placed in the upper left corner of the stand approximately 15 cm from the mouse. Participants kept their right hand on the mouse and when queried to respond, made their response using this hand. As soon as participants started to move the mouse, a cursor, identical in appearance to the discs, appeared at the centre of the screen and participants moved it to the perceived final position of the queried target, clicking the left mouse button at this location to register their response and to trigger the feedback. Feedback was given by displaying the queried target in the position that it had occupied just before its disappearance, i.e. the veridical final position. Both the veridical final position of the queried target and mouse click responses were recorded to the nearest display pixel. The feedback disappeared either after 1130 ms or when the participant pressed the keypad with their left index finger (whichever occurred sooner) to initiate the next trial. In the mouse click condition, participants held their left hand straight down the side of their body except when pressing the keypad to trigger the next trial.

In the case of pointing trials, participants were instructed to hold their left arm straight down the side of their body with the mouse in their left hand. With their right hand, participants were required to start each trial holding a key down on the keypad with their index finger until they were prompted to make their pointing response. When queried for a response, participants lifted the index finger of their right hand from the keypad and pointed at the perceived final position of the queried target with the instruction to ‘point directly at where you think the disc was at the final moment before it disappeared’. No instruction was given about how far from the screen the finger should be at the end of the pointing trajectory except that they should avoid touching the screen. Participants were instructed to click the mouse using their left hand at the moment they felt they were pointing directly at the final position of the queried disc. This triggered the appearance of the feedback disc, which remained on screen either for 1130 ms or until the participant returned their right index finger to the keypad and held the key down, which then triggered the next trial.

Feedback was given as soon as the response was registered and appeared in the form of the queried disc in its veridical final position. Feedback remained visible until either 1130 ms had elapsed or until participants pressed the keypad button, whichever was the earlier event. This keypad press then triggered the next trial after an ITI of duration randomly selected between 1420 and 1840 ms.

## Analysis

### Position monitoring performance

On every trial, we calculated the error magnitude, which is the distance in degrees of visual angle between the reported final position of the queried target and its veridical final position, meaning that good performance would be associated with small error magnitudes and vice versa. We also checked for any participants whose mean error magnitude was poorer than two standard deviations above the group mean in any condition and excluded them from further analyses in the condition for which these poor performance levels were identified. This performance threshold led to one participant’s data being excluded from each of the two response type conditions.

To check for possible floor effects, we first compared performance against what would be expected if participants were unable to perform the task. We simulated guessing performance by calculating the simulated magnitude of errors on every trial had the participant clicked at the centre of the area containing the queried target We analysed these data using a 2 (mouse click, pointing) × 4 (loads 1–4) ANOVA.

We examined the performance data for any evidence of hemispheric independence as proposed by Alvarez and Cavanagh (2005) by comparing performance in the two-target condition between trials on which the two targets were arranged unilaterally versus when one appeared in each hemifield (bilateral presentation). We also examined the effect of target final eccentricity on performance (as previously reported for pointing responses by Prablanc et al. 1979) by computing correlation between distance of the queried target from fixation at the time of its disappearance and error magnitudes for mouse click and pointing conditions. We expected more peripheral targets to be pointed at with less accuracy than targets occupying a more central final position.

### Perceptual lag versus extrapolation

In addition to error magnitudes, we calculated perceptual lags as previously reported by Howard and colleagues (Howard and Holcombe 2008; Howard et al. 2011, 2017). To calculate perceptual lags, a series of comparisons is made between the reported position of the queried target and the positions it occupied at increasingly old frames in the display leading up to its disappearance, as well as at extrapolated hypothetical future positions had it continued moving with the same speed and in the same direction of motion as its final velocity would dictate. Just as the mean error magnitudes represent the mean distance between the reported position and the veridical final position of the queried target at the moment before it disappeared, perceptual lag analyses compare reported positions with a range of past and future positions of the queried target. The time at which these positions of the target best resemble reported positions is found by localising the time on the curves at which these spatial differences are minimised, and this time of closest agreement is the perceptual lag value. The perceptual lag can be negative if reports most closely represent extrapolated future positions.

We also calculated angular errors as previously reported by Iordanescu, Grabowecky and Suzuki (2009) and Howard et al. (2011) as an alternative method for assessing the extent to which participants keep up-to-date representations of moving targets or even whether representations anticipate near-future positions. This method differs from the perceptual lag analysis in that it focusses on the headings of errors, rather than the times in the display that they best match. On each trial, the angular error is the angle subtended by the reported final position compared with the forwards vector of the queried target at the moment it disappeared. In this analysis, any responses lying directly ahead of the final motion trajectory of the queried target would possess an angular error of 0°. Responses lying perfectly along the opposite vector (pointing backwards) along the final motion trajectory would possess an angular error of 180°. All other intermediate angular errors represent varying similarity to the final trajectory of the queried target: for example, an angular error of 18° would mean that the participant reported a position anywhere along a line oriented 18° away from directly forwards. We calculated the fraction of trials in which responses lay anywhere between 0 and ± 90°, i.e. more forwards than backwards relative to the final motion vector of the queried target. Perceptual lag data and angular error data were each interrogated using a 2 (mouse click, pointing) × 4 (loads 1–4) ANOVA.

### Pointing trajectories

Using the data from the motion trackers (recording in millimetres), for each trial we calculated the speed of the index finger in the moments leading up to the end of the point. These data were first smoothed with a 10-ms moving window (which computed a sliding average) and then normalised by the total distance moved in the trial, so as to weight each trial equally in the trajectory analyses, rather than allowing larger magnitude points (for example when pointing near the top of the screen) to disproportionally affect the results. All subsequent pointing trajectory analyses are reported on these smoothed, normalised data. The statistical differences in acceleration profiles are examined in more detail below.

We calculated the time of the 10% threshold, which is the time that had elapsed before speed reached 10% of its final maximum on each trial. We also calculated the time of the speed inflection point where acceleration reversed into deceleration, and then calculated the fraction of time spent decelerating out of all the of post-threshold movement period. This measure of difficulty represents the time taken to complete the final stage of the pointing response as corrections are made at progressively finer spatial scales, until the pointing response is complete. For each load above 1, we compared this fraction against that observed under a baseline of one target. Each of these independent comparisons offers an assessment of the extent to which the kinematic profile in this specific condition differed from this baseline. We expected that this measure of pointing difficulty would reveal more difficulty pointing to the queried target at lower loads than higher loads, since lower loads are associated with greater internal representation precision, therefore effectively rendering the pointing target as a smaller, more precise area in space. These effects on the motor output might be expected to show up to a greater extent for more load-bearing dimensions (inferosuperior, and to a lesser extent, anteroposterior) of the pointing movement. Somewhat akin to a reaction time measure, we also analysed differences between load conditions for movement time before reaching the 10% speed threshold for each dimension. For each participant, we also calculated the Euclidian speed profiles in three-dimensional space. To assess differences in the early period of this three-dimensional acceleration where the pointing trajectory can be adjusted at the coarser spatial scales, we examined differences in speeds at each time sample using t-tests between pairs of loads.

### Postural sway

Given that participants performed the task from an upright stance, the possible effects of the task on posture control were also considered. Previous research has found that body sway increases in response to attention load during other cognitively demanding tasks such as visual search (Mitra 2003; Mitra and Fraizer 2004; Fraizer and Mitra 2008). Therefore, in the current work, it was a possibility that participants may exhibit different postural responses at different attention loads, which may in turn affect the precision of the pointing movement itself. To factor in these possible effects in our interpretation of results, we recorded body sway via a set of markers attached to the hip segment. Specifically, we were interested in, and concerned to take into account, any effect of attention load on sway produced during the execution of the arm movement itself. The motion tracking recording from the marker placed on the hip recorded the three-dimensional position of the hip in the room whilst participants made their pointing responses. We examined the final 3 s of recordings of positions of the hip leading up to the pointing response in the mediolateral and anteroposterior dimensions. Using a 100-ms sliding window, we evaluated standard deviation of positions within each window, and the mean value of this measure of variability for each participant as the window was applied over this period. Any effect of load on this postural maintenance measure was then assessed using ANOVAs (1, 2, 3 or 4 targets) in each of the two load-bearing (anteroposterior and inferosuperior) dimensions.

## Results

### Position monitoring performance

**Fig. 2:**
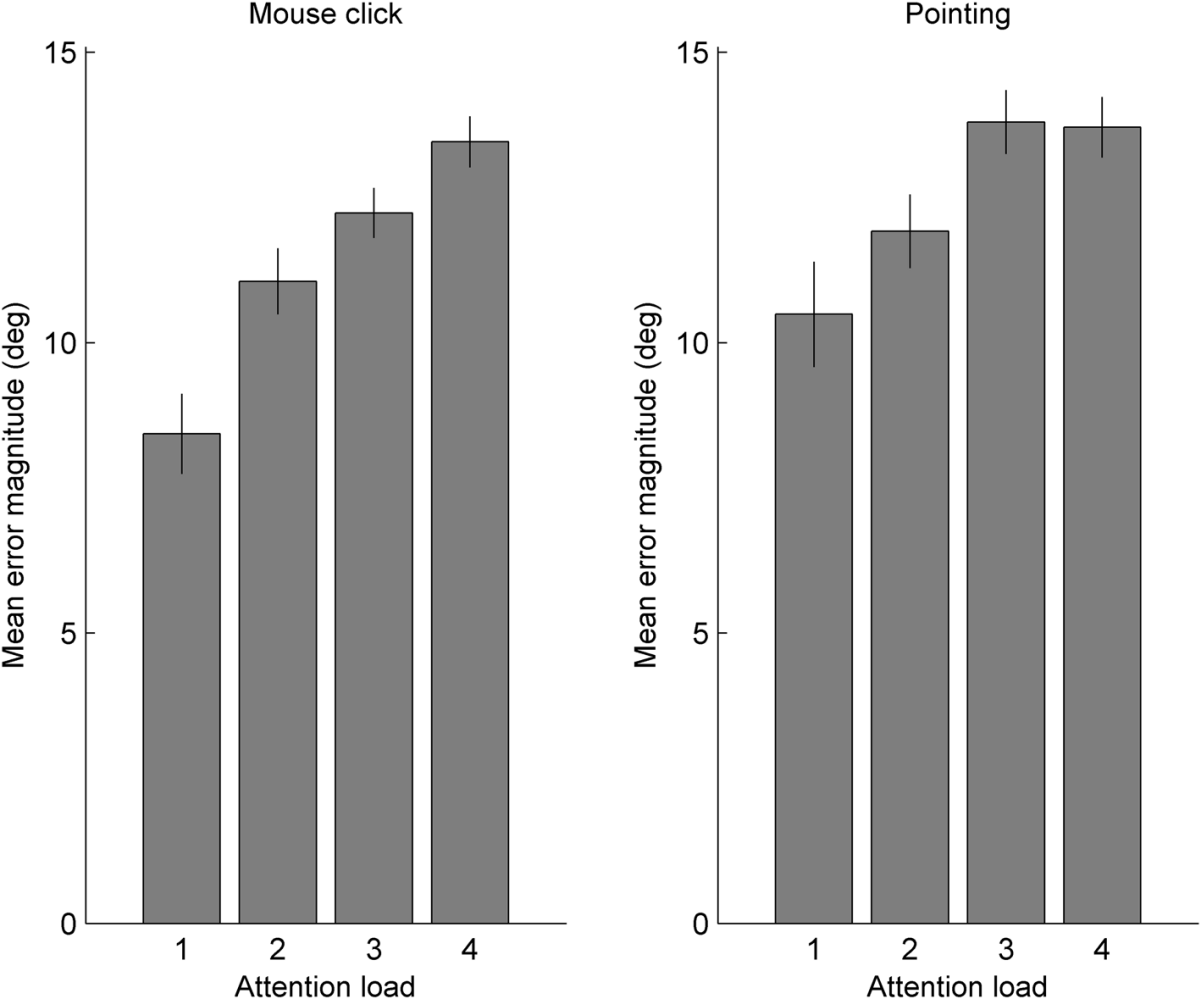
Mean error magnitudes for the two response type conditions under varying attention loads. Error bars indicate standard errors

**Fig. 3:**
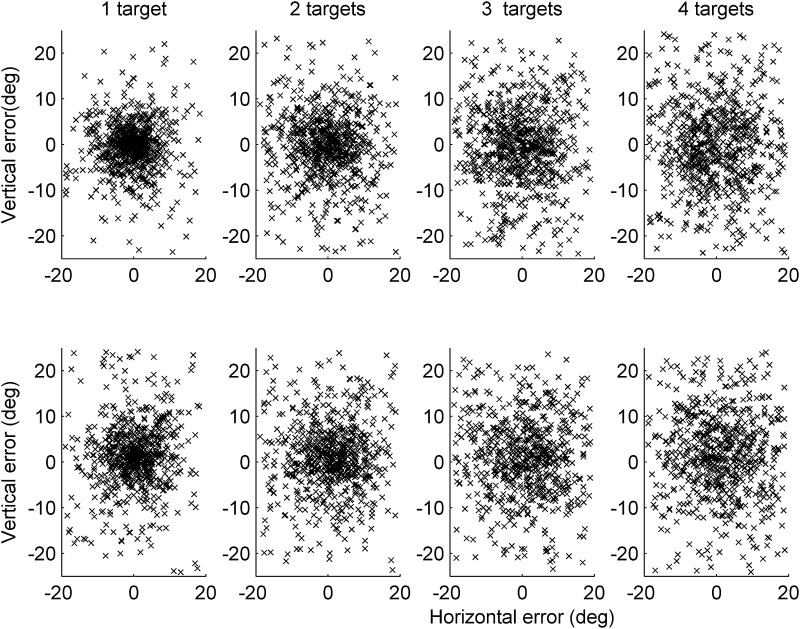
Horizontal and vertical error dispersions (relative to the display plane) for the four attention loads in the mouse click condition (top) and pointing condition (bottom), spatially locked to the veridical final position of the queried target at the origin (0,0). Note that the mean distance between responses and the origin are captured by the mean error magnitudes depicted in Fig. 2

Overall for mouse click trials, the mean error magnitude was 10.98° (SD 2.21). For pointing responses, the overall mean error magnitude was 12.11° (SD 2.71). For each attention load in both response type conditions, mean errors were significantly better than simulated guessing performance (all *p* < 0.001), ruling out floor effects in any individual load in either response type condition. As shown in Fig. 2, error magnitudes were greater in the pointing condition than the mouse click condition [*F*(1,24) = 11.08, *p* = 0.003, *η*_p_^2^ = 0.316] and tended to increase with attention load [*F*(3,72) = 52.11, *p* < 0.001, *η*_p_^2^ = 0.685]. There was a greater effect of attention load in the mouse click than the pointing condition, shown by the interaction between these two variables [*F*(3,72) = 2.53, *p* = 0.046, *η*_p_^2^ = 0.095]. This interaction (load × response type condition) was evident in differences between attention loads in each response condition—for mouse clicks, each addition to attention load produced successively poorer performance, but this was not true for pointing responses. For mouse clicks, every addition to the load caused a decrement in performance (all *p* < 0.001, highly significant even after using a corrected *p* value cutoff of 0.017 for three load-pair comparisons). The same was true for performance in the different pointing conditions for loads up to three (all *p*s < = 0.003) but monitoring three produced very similar performance to monitoring four targets (*p* = 0.958). Comparison with simulated guessing performance described above rules out floor effects as a possible source of this interaction between response type and attention load. Figure 3 illustrates these effects in terms of dispersion of responses around the veridical final position of the queried target.

For mouse click trials, mean errors did not differ between unilateral (mean 11.15°, SD 2.69) and bilateral (mean 10.37°, SD 2.45) trials [*t*(25) = 1.50, *p* = 0.15]. The same was true for pointing trials: unilateral (mean 11.31°, SD 3.61) and bilateral (mean 11.54°, SD 2.65) trials [*t*(25) = 0.42, *p* = 0.675] yielded similar performance. We also found a negative relationship between distance from the fixation point and performance (such that more central targets were responded to more accurately) in the mouse click [*r*(3437) = − 0.04, *p* = 0.037] but not the pointing [*r*(3437) = 0.01, *p* = 0.447] condition.

### Perceptual lag versus extrapolation

**Fig. 4:**
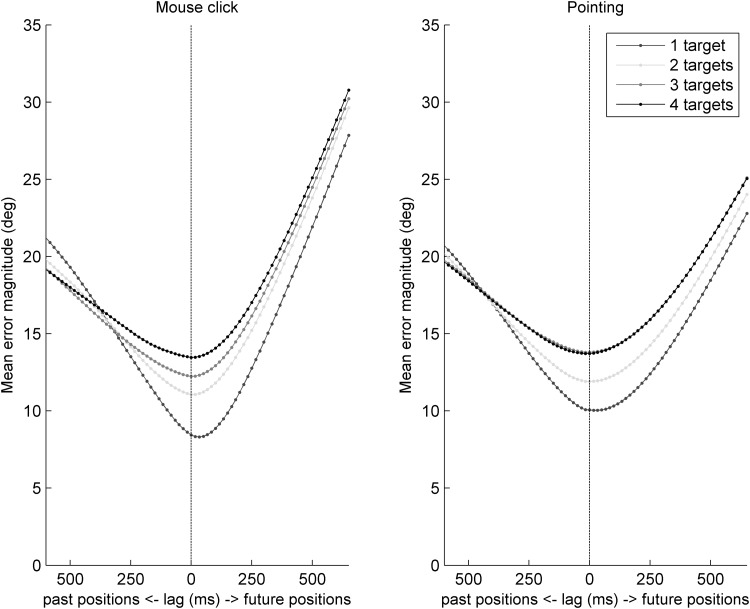
Perceptual lags in the two response type conditions and varying attention loads

Perceptual lag analysis results are shown in Fig. 4. The vertical dotted line represents the time at which the target disappeared (time zero); the points where the curves cross this line represent the mean error magnitude, that is, the mean distance between the reported final position of the queried target and the veridical position of the queried target on the last screen refresh of its presentation before it disappeared. The points on the curves immediately to the left of the dotted line represent the mean difference between the reported final position of the queried target and the position it occupied one screen refresh (~ 17 ms) before the final refresh of its presentation. Points progressively further to the left of the plots represent the mean difference between the reported positions and positions that the queried target had occupied at successively further moments from the past. Points to the right of the plots are the mean differences between the reported positions and the positions the queried target would have occupied had it continued moving in its final trajectory for progressively longer periods extrapolated into the future.

For mouse click trials with one target, perceptual lags were of magnitude − 26.28 ms (or equivalently, 26.28 ms extrapolation, SD 34.70). Perceptual lags were − 2.56 ms (SD 52.01) for monitoring two targets, 19.87 ms (SD 94.87) for monitoring three targets, and 14.74 ms (SD 65.38) for monitoring four targets. These differed significantly from a mean magnitude of zero only for one-target trials (*p* = 0.009, significant even using a corrected *p* value cutoff of 0.013); lags did not differ from zero for the other load conditions (*p* ≥ 0.261). Similarly for pointing trials, perceptual lags were − 28.67 ms (SD 45.52) for one target, − 3.21 ms (SD 60.19) for two targets, 10.26 ms (SD 62.73) for three and 19.87 ms (SD 70.24) four targets. These differed significantly from a mean magnitude of zero for one-target trials (*p* = 0.004, significant even using a corrected *p* cutoff value of 0.013) but not for any other loads (*p* ≥ 0.162).

Overall there was no effect of response type on perceptual lags [*F*(1,24) = 0.20, *p* = 0.660, *η*_p_^2^ = 0.008] but there was an effect of attention load on lags [*F*(3,72) = 4.70, *p* = 0.008, *η*_p_^2^ = 0.164] and no interaction [*F*(3,72) = 0.08, *p* = 0.972, *η*_p_^2^ = 0.0003]. Overall there was a trend for greater extrapolation (or equivalently, less perceptual lag) under lower attention loads.

### Forwards versus backwards angular errors

For mouse click trials, this analysis yielded forwards-biased angular error as significantly more likely than chance (50%) for all attention loads. For monitoring one target, 61.69% (SD 10.35%) of trials were more forwards than backwards biased, for monitoring two, three and four targets these fractions were 54.28% (SD 10.33%), 52.08% (SD 10.89%) and 52.44% (SD 7.77%), all significantly different from 50% as would be expected from no overall forwards or backwards tendency (all *p* < 0.001, highly significant even after using a corrected *p* value of 0.013 for four comparisons). For pointing trials with one target, mean forwards-biased angular errors were significantly less frequent than chance at 48.38% (SD 12.81%, *p* < 0.001). For two, three and four targets, these fractions were more forwards than backwards at 52.66% (SD 12.61%) for monitoring two, 52.78% (SD 12.02%) for monitoring three and 51.85% (SD 14.43%) for monitoring four and in all cases these were significantly so (*p* < 0.001, even after using a corrected *p* value of 0.013).

For these proportions of forwards versus backwards errors, there was no effect of response type on the fraction of forwards responses [*F*(1,24) = 2.19, *p* = 0.151, *η*_p_^2^ = 0.078], nor attention load [*F*(3,72) = 1.51, *p* = 0.220, *η*_p_^2^ = 0.055], but there was an interaction [*F*(3,72) = 8.50, *p* < 0.001, *η*_p_^2^ = 0.246]. This interaction was driven by more effect of response type at attention load of one target than at higher loads, since angular errors were more forwards-biased in mouse click trials and more backwards-biased in pointing trials for loads of one [*t*(26) = 4.09, *p* < 0.001, *d* = 0.79; *p* ≥ 0.598 for all other pairs for higher attention loads].

It is important to note that the type of analysis used can be critical in assessing whether perception most resembles predicted future positions or recent past positions: here we see responses tending towards the future for one-target trials in both response conditions using perceptual lag analyses, whereas in angular error analyses we see responses tending towards the future in all cases except one-target pointing trials, where responses tended towards the past. This seeming incongruity arises because the two analyses differ in the relative weights given to responses of different spatio-temporal and angular magnitudes. For example, in the angular analysis, if one response lies just ahead of the final position of the queried target and another lies substantially far behind, these two would be given equal weighting in the overall result. They would not, however in the perceptual lag analysis, which would weight the latter response more heavily since it is of greater spatio-temporal magnitude. Although an overall trend is not clearly apparent between analyses, it is not the case that increasing engagement of the motor system in the pointing condition allows participants to better compensate for any lagging processes. In the angular error analysis, pointing reports actually resembled the past more than the future where mouse click responses tended to resemble the predicted near future.

### Pointing trajectories

**Fig. 5:**
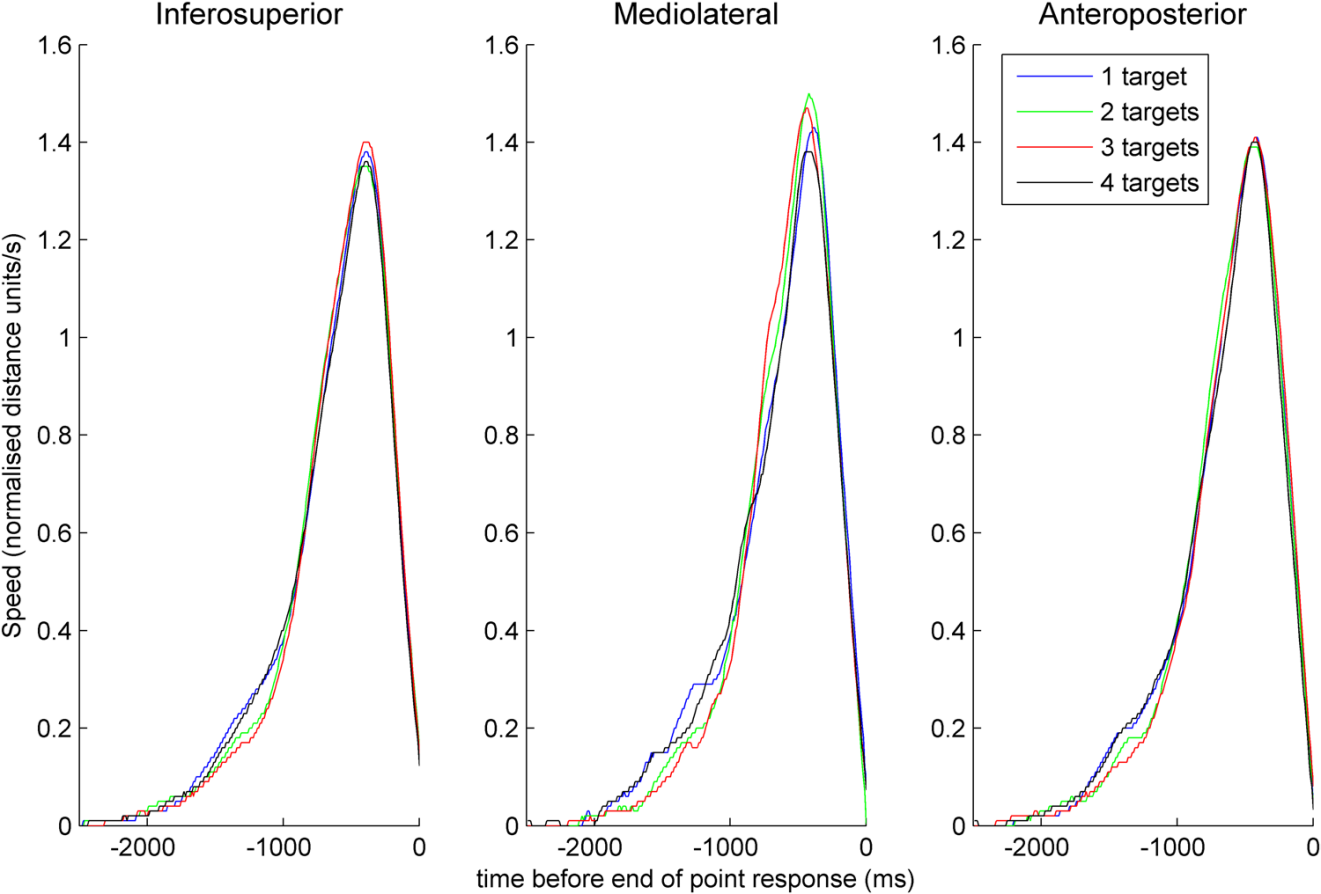
Normalised speed profiles in the moments leading up to the end of the pointing response across the three spatial dimensions relative to the body and across attention loads

Numerically, in all three spatial dimensions relative to the body, it is apparent that both the lowest and highest loads (i.e. loads of one and four targets) are associated with an early and short-lived acceleration (around 2000–1000 ms before the trajectory endpoint) relative to the other load conditions (two, and especially three targets), after which we see in all four loads the main period of acceleration towards, and then deceleration away from peak speed leading up to the trajectory endpoint at time zero (Fig. 5 shows these speed profiles).

In terms of the fraction of time spent decelerating out of the of post-threshold movement period, for the inferosuperior dimension this fraction was 64.90% (SD 10.80%) for monitoring one target, 64.34% (SD 11.05%) for monitoring two, 63.17% (SD 10.82%) for three and 64.45% (SD 10.68%) for monitoring four targets. These fractions did not differ between loads of one and two targets [*t*(26) = 0.75, *p* = 0.462, *d* = 0.14] or between loads of one and four targets [*t*(26) = 1.10, *p* = 0.282, *d* = 0.21] but were different between one and three targets [*t*(26) = 2.86, *p* = 0.008, *d* = 0.55, and this comparison remained significant after correction for three comparisons using a *p* cutoff of 0.017].

However for the mediolateral dimension these fractions did not differ between any loads: 69.37% (SD 17.08%) for monitoring one, 70.38% (SD 15.41%) for monitoring two, 69.06% (SD 16.21%) for monitoring three and 69.04% (SD 15.96%) for monitoring four targets. These fractions did not differ between loads of one and two [*t*(26) = 0.90, *p* = 0.376, *d* = 0.17], between one and four targets [*t*(26) = 0.37, *p* = 0.716, *d* = 0.07] nor between one and three targets [*t*(26) = 0.38, *p* = 0.704, *d* = 0.07].

Like the inferosuperior dimension, these fractions in the anteroposterior dimension were significantly different between monitoring one and three targets but not between other load comparisons: 67.92% (SD 11.24%) for monitoring one target, 67.38% (SD 12.04%) for monitoring two targets, 66.34% (SD 12.37%) for monitoring three targets and 67.87% (SD 11.47%) for monitoring four targets. These fractions were not different between loads of one and two targets [*t*(26) = 0.90, *p* = 0.375, *d* = 0.17] or between loads of one and four targets [*t*(26) = 0.08, 0.941, *d* = 0.02] but were different between one and three targets [*t*(26) = 2.42, *p* = 0.023, *d* = 0.47, however this comparison is larger than required for a corrected *p* cutoff of 0.017]. It is of note that the difference in this metric between loads was apparent in the most load-bearing inferosuperior dimension and approaching significance in the second-most load-bearing, anteroposterior dimension. In terms of movement time before reaching the 10% speed threshold, for the inferosuperior dimension, no pair of loads were significantly different from one another (in all cases *p* ≥ 0.331). For the mediolateral dimension this was also the case (in all cases *p* ≥ 0.053) and similarly for the anteroposterior dimension (in all cases *p* ≥ 0.301).

**Fig. 6:**
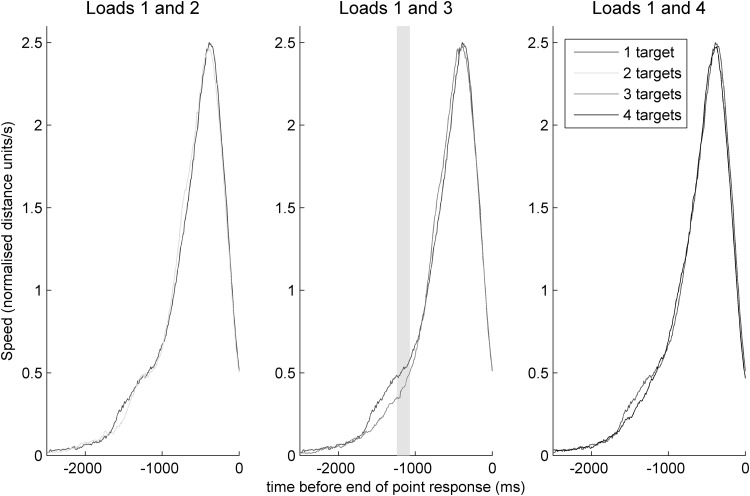
Mean Euclidian speed profiles in three-dimensional space for comparisons between load conditions. Blue lines indicate trials with a load of one, green lines indicate a load of two, red indicates a load of three and black indicates a load of four. The left and right panels show an early interval of slightly (though not significantly) greater acceleration for trials with a load of one compared to loads of two (left panel) or four (right panel) targets. The central panel shows a sustained early period (particularly ~− 1250 to ~− 1000 ms) in which trials with a load of one target are associated with significantly greater early acceleration than three-target trials

Similar to the individual dimension speed profiles in Fig. 5, the Euclidian speed profiles in three-dimensional space (Fig. 6) reveal an early and relatively short-lived period of acceleration in the high and low loads compared to intermediate loads (see comparison between loads of one and three targets in the middle panel; significant differences where *p* < 0.0167 between loads are shown in grey). This contrast appears to indicate an early interval of ongoing trajectory error correction for these high and low loads between approximately 1500 and 1000 ms before the trajectory endpoint. There were no significant differences in peak speeds, as shown by the absence of grey areas at the peaks of trajectories.

### Postural sway

In the anteroposterior dimension, for monitoring one target, the mean of the postural variability was 6.30 mm (SD 3.93 mm), for monitoring two it was 6.89 mm (SD 4.57 mm), for monitoring three it was 7.03 mm (SD 5.93 mm) and for monitoring four it was 7.63 mm (SD 7.10 mm) showing no overall effect of load [*F*(3,78) = 2.21, *p* = 0.166]. In the mediolateral dimension, for monitoring one target, the mean of this variability was 4.21 mm (SD 2.91 mm), for monitoring two it was 4.42 mm (SD 3.37 mm), for monitoring three it was 4.73 mm (SD 4.79 mm) and for monitoring four it was 5.23 mm (SD 5.80 mm) showing no overall effect of load [*F*(3,78) = 2.12, *p* = 0.158].

## Discussion

Both response conditions reveal a general attention load effect consistent with previous results (Howard and Holcombe 2008; Howard et al. 2011) and a gradual decrease in precision with each increment in load in the mouse click condition. Further, in the pointing condition, performance does not differ between the three- and four-target conditions, and this equivalence is shown not to be a floor effect. This pattern of performance is consistent with reduced distractor inhibition in the pointing condition. If in the pointing condition, under the higher loads, participants cannot effectively inhibit processing of the distractor objects, then they may be processing all four objects in both the three- and four-target conditions, leading to the same level of representational imprecision in both these conditions and thus to exactly the pattern of performance observed here. This lack of load effect between monitoring three and four targets is not consistent with an account based on more limited capacity in the pointing condition than the mouse click condition. Rather, a reduction in capacity would cause an even more dramatic reduction in performance at loads of four compared to three targets. For example, if people could only attend to a maximum of three objects in the pointing condition, then in the four-object condition, they would be successfully attending to three objects, so would perform at the same level as the three-target condition on three-quarters of trials and would be guessing on the other quarter of trials, leading to worse overall performance than is seen in the three-target condition. However, if participants cannot effectively inhibit attention to distractors, then we would see a flattening of the set size effect, just as we report here. In traditional MOT tasks where targets and distractors regularly cross each others’ paths, distractors have more ability to draw attention away from targets than they do in the display used here. However, distractors are still likely to impair performance here since they are luminance-defined objects moving in the display, and salient moving objects are known to attract attention (Abrams and Christ 2003; Franconeri and Simons 2003, 2005). Such difficulty in fully suppressing attention to distractors seems plausible since it is known that distractor suppression is an active process requiring attentional resources (Belmonte and Yurgelun-Todd 2003; Bettencourt and Somers 2009; Pylyshyn et al. 2008). One reason that we may see this reduced distractor suppression for the pointing condition is that hand position, posture and reachability of objects have been shown to facilitate visual processing: Thomas (2013, 2015, 2017) has shown that placing hands near the display enhances visual processing, especially when the hand is in a posture appropriate for the response, as was the case here, in that the index finger was placed ready on the keypad in preparation for the pointing response (also see Reed et al. 2010; for a review see; Brockmole et al. 2013). It may be that reachability of visual displays automatically prompts the preparation of motor plans, which necessarily enhances processing of some aspects of the stimuli, such as distance from the hands, shape, etc. Perhaps this reachability inappropriately facilitated visual processing for all objects, especially when distractor suppression mechanisms were most under-resourced. This explanation in terms of motor spatial interference with visual spatial distractor suppression offers an avenue for future behavioural and cognitive neurophysiological research on interactions between body position and motion tracking.

In terms of performance levels we did not see a facilitation of the monitoring task by increasing engagement of the motor system. In fact, pointing precision was overall slightly worse than that of mouse click responses. This result, the inverse of our hypothetical prediction, is likely due at least in part to uncertainty around whether or not the to-be-reported position is defined by a line extending out of the participant’s finger and intersecting with the screen, or whether it is the position on the screen occluded by the end of the finger. Another source of uncertainty is in eye dominance, since participants will vary in whether the right or left retinal image is given more weight in this alignment task (e.g. Porac and Coren 1975). Variability will also arise during the brief moment the finger is suspended in its point. These possible additional sources of noise in the pointing condition may also have obscured any effects of eccentricity of the final position of the target. However, we find no evidence that increased engagement of the motor system facilitates performance. Both mouse click and pointing responses were made after the stimulus had disappeared and hence were relatively offline compared, for example to interception tasks. However, the pointing response was still relatively online in the sense that ongoing adjustments to the pointing trajectory could be made by using visual and proprioceptive feedback about the current position of the arm and hand relative to the representation of the queried target’s position. It is possible that we would have seen facilitation of the monitoring task under the pointing condition had the task been performed online during the motion phase, for example, if participants had been asked to manually intercept targets. However, here we were interested in the way that responses are directed to an internally represented position, hence the pointing responses were prompted after target disappearance. We find no evidence for hemispheric independence since performance was comparable when two targets were tracked in two hemifields or one. There is a possibility that our instruction to respond with the right hand heightened reliance on left-hemispheric resources; however, this inference cannot be established on the basis of these findings.

Involvement of the motor system through requiring pointing responses did not significantly affect perceptual lags of the type described by Howard and colleagues (Howard and Holcombe 2008; Howard et al. 2011, 2017). In both response conditions, we report negative perceptual lags (i.e. slight extrapolation) for monitoring a single target. When we calculated angular errors as described by Howard, Masom and Holcombe (2011) and Iordanescu et al. (2009) although there was no effect of the pointing task compared to mouse click responses, there was an interaction between type of response and proportion of forwards responses, with mouse click responses producing extrapolated reports and pointing producing lagging reports for monitoring one target. These two analyses appear to produce divergent results on the issue of lag versus extrapolation, since trials on which the angular error is even slightly biased in the forwards direction will be weighted the same as trials on which clear backwards angular errors are observed, in contrast to the perceptual lag analysis which weights trials on the grounds of the time that they best resemble. In this case, perceptual lag analyses produce lagging results, whereas the angular errors produce results weighted towards forwards errors. Perceptual lag analysis yielded similar results between response type conditions. However, in the angular error analysis, contrary to what was predicted, pointing actually increased backwards responses compared to mouse click trials. When objects are nearer the hands, it has been shown that there is biasing away from high spatial frequency information in the parvocellular system and towards high temporal frequency in the magnocellular pathway (Goodhew et al. 2013; Gozli et al. 2012). This bias could potentially explain the poorer overall spatial precision seen in the pointing condition. It may also have encouraged participants to favour a strategy of attention to moment-by-moment changes in position, rather than using other strategies such as representations based on speed, direction or other specific motion-based characteristics. This explanation would be consistent with previous work showing that slight differences in task instructions can modify lags—attention to changing position rather than attention to the motion of moving targets yields different lags (Howard et al. 2017). Therefore, greater attention to position as it changes over fine temporal scales in the pointing task may have modified the extent to which responses resemble past versus near-future positions of targets.

By manipulating the number of attended targets, we tested how the number and precision of internally represented positions affect the motor response. We report effects of load on kinematics both in terms of the fraction of the trajectory spent in deceleration after the 10% speed threshold had been reached, and in terms of times during the trajectory at which speeds differed between loads. For the fractions of time spent in deceleration, we see a difference between monitoring one and three targets in the most load-bearing, inferosuperior dimension and a trend in this direction in the second-most load-bearing, anteroposterior dimension. Greater early adjustments for more load-bearing dimensions would be an adaptive motor strategy because load increases as the hand travels further from the body, and adjustments therefore become more metabolically costly the later they occur during the point. For the speed comparisons across time points in the trajectory, we see a sustained period early in the trajectory where speeds likewise differed between loads of one and three targets. Therefore, across both speed and deceleration measures, different processes seem to operate at the intermediate load of three targets compared to lower and higher load conditions. Why would we see this non-monotonic relationship between load and trajectory characteristics, in other words, why would the highest load (four targets) and lowest loads (one target, and perhaps to a lesser extent, two targets) produce similar trajectories while the intermediate load of three targets is associated with a different pattern? We did not, a priori, expect to see such a relationship and our experiment was not designed in order to answer this question. However, we suggest an account below to explain these findings.

In the task presented here, there are two ways in which load can affect processing after the display has offset and the participant prepares their pointing motor response. The first mechanism by which load will affect these post-perceptual processes is in the precision of the internal representation of the queried target, and the second is by competition between representations of targets. We argue that these two mechanisms produce two separate sources of difficulty for the motor system: the difficulty of aiming a pointing response towards a very finely represented location and the difficulty of producing a pointing response when no-longer-relevant competing representations must be discarded. The first source of difficulty is greatest at lower loads and the second is greatest at higher loads.

We turn first towards the effect of representational precision on pointing responses. It has previously been shown that increases to the number of targets for attentional monitoring decreases the precision with which their positions are represented (Howard and Holcombe 2008; Howard et al. 2011, 2017). Equivalently, the fewer the targets, the greater the precision of the representation of their positions. Classic Fitts’ Law (1954) effects would predict that more difficult pointing tasks would show greater adjustments for targets that dictate higher levels of pointing precision, for example when pointing to small physically present targets. Here we show this relationship to be true not just when precision varies according to a real physical target, but for the precision of an internal representation of the target position, with more adjustment for more precisely represented targets.

Secondly, our finding that high loads evoke similar periods of high early adjustments as do low loads (compared to intermediate loads), suggests that the number of competing internal representations also contributes to the extent of these adjustments. In the task presented here, after monitoring a single-target participants needed only to aim their pointing response at this single, precisely represented position. With two targets, although only one of the two targets for monitoring was queried, participants needed to select the correct representation, keeping it distinct from the other, no-longer-relevant representation, and to use this selected representation to guide their pointing response. This representation would also be less precise than would be the case for single-target trials. Accordingly, for three- and four-target trials, representations would become progressively less precise and selection of the queried target representation would involve discarding increasing numbers of no-longer-relevant alternative representations, whilst keeping the one used to guide pointing distinct from the others. We suggest that the additional demands to keep the representation of the queried target distinct from those of no-longer-relevant non-queried targets can explain the apparent difficulty in pointing responses for higher loads (in this case, four-target trials).

We found a non-monotonic relationship between attention load and the extent to which we see an early acceleration period followed by a period of reduced acceleration. This was the case both when using a metric based on the proportion of deceleration time as a fraction of post-threshold durations, and as a simple difference in speed across time points. These early acceleration periods, followed by reduced acceleration, appear to reflect early adjustments made during the pointing trajectory. One-target and four-target trials were associated with similarly great adjustments compared to intermediate loads. Although differences between loads of one and two targets were not statistically significant, they were in the same direction numerically as the significant differences seen between loads of one and three targets. Therefore, these data suggest that the temporal dynamics of precision motor responses are driven not only by the demands of the physical environment, but by the quality of internal representations of the environment.

For experiments using a single static target, some have shown that ongoing corrections to the trajectory can be made to account for discrepancies between the internal target representation and visual information about the current position of the response limb (Heath 2005) though other studies show only limited adjustments during memory-guided reaching (Heath et al. 2004). Adjustments also appear to be made to correct for accumulated neuromotor noise (e.g. Medina et al. 2009; Meyer et al. 1988). The findings we present here add to the factors known to feed into the ongoing kinematics of motor responses.

Few studies have investigated motor responses where multiple targets are involved, however Elliott and Calvert (1990) compared simple and choice responses. These were comparable to our one- and two-target conditions since in either case one response was required, and the queried target identity was either known or unknown during stimulus presentation, respectively. These authors removed visual information about the stimuli at initiation of participants’ responses therefore forcing a reliance on internal representations. They reported little difference between simple and choice performance in terms of accuracy, movement times and reaction times. In contrast, Hansen et al. (2006) similarly compared simple versus choice responses in a number of conditions, including conditions of limited visual information, and reported differences in reaction times but not movement durations between simple and choice trials. However, in the Hansen et al. (2006) study, a high degree of precision was not required, since targets were relatively large at 2.5 cm in diameter. In both of these simple versus two-choice response studies, the effect of multiple representations is investigated only to a limited degree, since at most two representations are involved. Thus, the data that we present here extend this investigation considerably. Further, to our knowledge, the additional demand of attending to changing positions over time has not been previously investigated. The fact that our participants were required to attend to these changing positions over a period of seconds adds greatly to the ecological validity of our task, since the real world is frequently dynamic in nature and individuals are only rarely presented with a truly static view of the environment.

In summary, we find that in position monitoring tasks, increasing the engagement of the motor system limits the extent to which distractors can be inhibited, and does not increase the ability of the visuomotor system to compensate for neural delays by extrapolating the positions of moving targets. In terms of the control of motor kinematics, attention load modifies the extent of early adjustments during the pointing trajectory, more adjustments being made for both very high and low loads. This high degree of trajectory adjustment is required to meet the demands of pointing towards precisely represented targets for very low loads, and to meet the demands of keeping the queried target representation distinct from competing no-longer-relevant representations for high loads.
